# What Can a Zebrafish See with Only an Off-Pathway and Other Fish Stories?

**Published:** 2012-01

**Authors:** John E Dowling

**Affiliations:** Department of Molecular and Cellular Biology, Harvard University, Cambridge, MA, USA

Zebrafish have a marvellous visual system for study. Their retinas have abundant cones of four spectral types, i.e. red-, green-, blue-and ultraviolet–sensitive cones. The animals are exceptionally responsive behaviourally; robust visual responses can be measured just four to five days after egg fertilization^1^. My group has been carrying out forward genetic studies on zebrafish for about fifteen years, seeking mutants with altered retinal neurons and/or circuitry such that we can advance our understanding of visual mechanisms. In addition, we study the functional organization of the retina in normal zebrafish. Herein, I describe two studies, one on a mutant that is providing new information on photoreceptor synapses and how certain visual responses may be mediated by the animal, and the other on an interesting phenomenon that occurs in young, larval zebrafish.

I began with the mutant designated nrc. To isolate behavioral mutants we screen mutagenized fish by observing the optokinetic reflex (OKR)^2^. This is simply performed by observing five to six-day-old fish placed in a petri-dish inside a rotating drum lined with black and white stripes. We observe whether a fish can move its eyes in response to the moving stripes. With this test we have found completely blind, partially blind, color blind and even movement deficient fish^1^. The nrc fish was discovered because it completely failed in the OKR test, under all tested conditions. The fish appeared entirely normal, even the retinal histology looked fine. Close examination at the light microscope level showed a thin outer plexiform layer but little else. Electron microscopy, on the other hand, revealed, that the photoreceptor terminals were strikingly abnormal.^3^ Most of the processes from bipolar and horizontal cells failed to penetrate into the terminals and consequently ribbon synapses in the terminals did not form. We did observe basal synaptic contacts, although they were displaced to the base of the terminals (Fig. 1).

Ribbon synapses drive ON-bipolar cells, whereas basal synapses drive OFF-bipolar cells, suggesting there are no ON-responding bipolar cells in the nrc retina, but perhaps some OFF-responding bipolar cells. This supposition was tested and confirmed by recording ERG’s from nrc retinas. The b-wave of the ERG reflects mainly ON-bipolar cell activity while the d-wave corresponds to OFF-bipolar cell responses. Although there was no or minimal b-wave activity recorded from these fish, d-waves were seen consistently. ERG d-waves suggest that OFF-ganglion cells may be activated in nrc retinas and this was shown to be so by recording from single ganglion cells.^4^ Robust OFF-ganglion cells were recorded, but no ON-cells. A surprise was that some ON-OFF ganglion cells could be recorded in these retinas, but the ON component of the response had long latency and was sluggish.

If there are light-driven ganglion cells in the nrc retina, why do these animals fail to show an OKR? One possibility is that they fail to see movement which is required to elicit an OKR. A novel behavioral test was devised to test whether nrc fish respond to light and dark transitions.^4^ Called the visual motor response (VMR), this test showed that nrc fish respond robustly to light–to-dark transition but only slowly and weakly to a dark-to-light transitions. The behavioral results matched well with the ganglion cell recordings. These results suggested that pure ON-cells are required by zebrafish to see moving stimuli and this was confirmed by pharmacologically blocking the ON-pathway in normal fish. In such fish the b-wave disappears but the d-wave remains. Furthermore, no pure ON-ganglion cells were detected in these retinas, but robust OFF-ganglion cells were readily recorded along with some ON-OFF cells whose ON response had long latency and was sluggish. As expected, these fish showed no OKR. We conclude that the ON-pathway is required by zebrafish to see movement (at least as far as the OKR is concerned), but the fish can detect light-dark transitions. Furthermore, we propose that the sluggish ON-component of the ON-OFF ganglion cells arises from the OFF-pathway, perhaps via sign-reversing amacrine cells.

The other study involves a robust circadian rhythm phenomenon displayed by young (larval), wild-type zebrafish. A few years ago we (Li and I^5^) demonstrated that in adult zebrafish, a circadian rhythm alters rod and cone sensitivity by up to two log units as a function of time of the day. During the night, zebrafish lose light sensitivity as measured by ERG, but regain it in the daytime. Surprisingly, examination of larval fish showed they essentially turn off retinal responses at night.^6^ Not only are ERG responses less sensitive, bright lights generate only tiny responses at night time when b-wave amplitudes are reduced by 95% or more. Behaviorally, the fish respond as if they are blind, failing both the OKR and VMR tests. It is possible to restore light responsiveness by illuminating the fish at night, but darkness during the day has no effect. By isolating the a-wave of the ERG, which arises from photoreceptor outer segments, we found a-wave amplitudes were reduced by about 60%, explaining part but not all of the loss of b-wave responsiveness. Interestingly, we observed that synaptic ribbons disappeared from the photoreceptor terminals at night, suggesting this might be another mechanism that reduces photoreceptor activity in the larval fish.

Why would zebrafish turn off their photoreceptors at night? It could simply be to save energy. The animals at this stage are just beginning to feed and they have exhausted their yolk which has sustained them until this point in life. Dark-adapted photoreceptors consume a considerable amount of energy to maintain themselves (10^8^ ATP/second in a single dark-adapted mouse rod), and eliminating this large energy drain could be critical at this time in the lives of these young fish.

These two stories illustrate the usefulness of zebrafish as a model organism for studying visual mechanisms (Fig. 2). Combining physiology with behavior coupled with genetics and fast development provides a powerful approach.

## Figures and Tables

**Figure 1.**
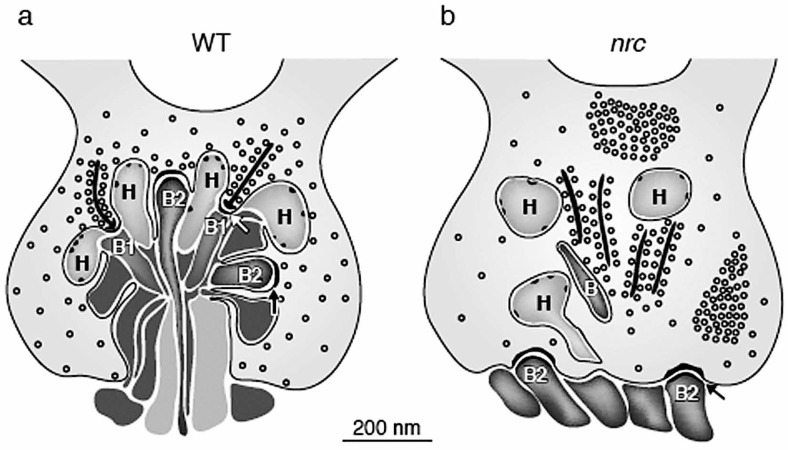
Diagram of photoreceptor terminals in wild type (WT) and nrc mutant zebrafish at 5 dpf. (a) In the WT retina, bipolar and horizontal cell processes invaginate into the pedicle in a tight bundle to make two types of synaptic junctions: ribbon synapses and basal contacts. Ribbon synapses are made onto presumed ON-bipolar (B1) and horizontal (H) cell dendrites (white arrows). Basal contacts, made on presumed OFF-bipolar cells (B2), are found between the ribbon synapses and have dense cytoplasmic material on both sides of the junction. (b) In the nrc retina, few processes invaginate into the photoreceptor terminals. However, when present, many of these processes have small membrane densities, characteristic of horizontal cell processes. Synaptic ribbons in most of the pedicles are unassociated with postsynaptic processes and appear to be floating. Basal contacts are seen onto presumed OFF-bipolar (B2) cell dendrites. However, they are displaced and make junctions along the photoreceptor base rather than within the photoreceptor terminal. Synaptic vesicles often clump and fail to distribute evenly in nrc pedicles, but they surround synaptic ribbons, as they do in WT pedicles.^7^

**Figure 2.**
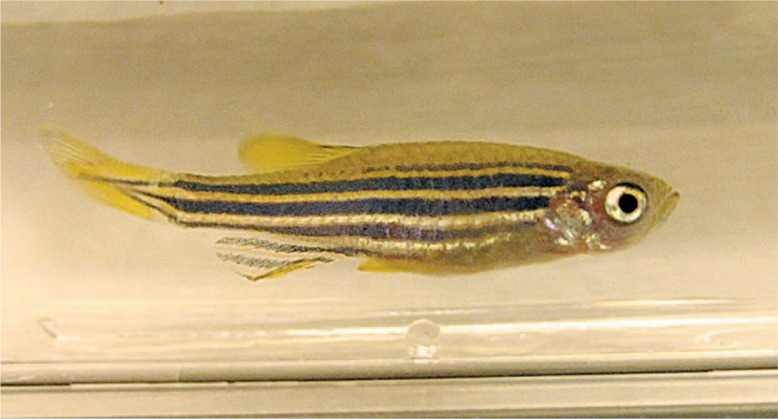
The zebrafish, Danio rerio, is an important vertebrate model organism in eye research.
